# Interplay Between Calcium and AMPK Signaling in Human Cytomegalovirus Infection

**DOI:** 10.3389/fcimb.2020.00384

**Published:** 2020-07-29

**Authors:** Diana M. Dunn, Joshua Munger

**Affiliations:** Department of Biochemistry and Biophysics, School of Medicine and Dentistry, University of Rochester, Rochester, NY, United States

**Keywords:** cytomegalovirus, HCMV, calcium signaling, AMPK, cancer, oncomodulation

## Abstract

Calcium signaling and the AMP-activated protein kinase (AMPK) signaling networks broadly regulate numerous aspects of cell biology. Human Cytomegalovirus (HCMV) infection has been found to actively manipulate the calcium-AMPK signaling axis to support infection. Many HCMV genes have been linked to modulating calcium signaling, and HCMV infection has been found to be reliant on calcium signaling and AMPK activation. Here, we focus on the cell biology of calcium and AMPK signaling and what is currently known about how HCMV modulates these pathways to support HCMV infection and potentially contribute to oncomodulation.

## Introduction

Human Cytomegalovirus (HCMV) infection is a prevalent opportunistic pathogen, infecting ~60–90% of the global population (Pass, 2001). It remains latent in most individuals, but causes significant morbidity in immunoimmature or immunocompromised hosts including neonates, transplant recipients, AIDS patients, and cancer patients undergoing immunosuppressive therapies (Pass, 2001; Kuo et al., 2008; El-Cheikh et al., 2013; Tay et al., 2013; Teh et al., 2013). Evidence of HCMV infection and its contributions to mortality in cancer patient populations has increased over time (Nguyen et al., 2001; Boeckh, 2011; Wang et al., 2011; Tay et al., 2013; Jaillette et al., 2016; Rådestad et al., 2018), partially due to inadequate anti-HCMV therapeutics. Approximately 0.6% of babies in developing countries are HCMV seropositive, and 10% of infected infants suffer from microcephaly, hearing and vision loss, mental impairments and even death, making HCMV infection one of the leading causes of birth defects (Schottstedt et al., 2010; Swanson and Schleiss, 2013). Current therapies often have poor bioavailability, exhibit long-term toxicity in afflicted patients and can often lead to the development of drug resistance due to the prolonged treatment regimens required to eliminate lytic infection (Weisenthal et al., 1989; Flores-Aguilar et al., 1993; Goodrich et al., 1993; Andrei et al., 2008).

HCMV can spread through mucosal membranes, via blood, through the placenta and breast milk from mother to child, or through saliva and sexual secretions. Primary infection and viral replication occur in a wide variety of cell types including fibroblasts, epithelial cells, endothelial cells, mononuclear cells, and neural progenitor cells (Sison et al., 2019). Viral latency is established in hematopoietic stem cells, as these cells differentiate into myeloid derived macrophages and dendritic cells, they become more permissive to reactivation of the virus (Hahn et al., 1998; O'Connor and Murphy, 2012; Forte et al., 2020).

HCMV is a betaherpes virus containing a large double-stranded DNA genome of about 230,000 base pairs (Schottstedt et al., 2010), encoding for a largely unknown proteomic potential (Stern-Ginossar et al., 2012). The regulation and function of these gene products by both the virus and host cell shape the viral environment and the potential to successfully produce viral progeny. It is known that the upstream calcium-calmodulin signaling cascade protein, CaMKK, and its downstream target, AMP-activated protein kinase (AMPK), both play a critical role in HCMV-mediated glycolytic activation and viral replication (McArdle et al., 2011, 2012). These two overlapping pathways were proven to be crucial for activation of glycolysis during infection, but both impact cellular function beyond metabolic regulation. In this review we will explore the contributions of calcium signaling and AMPK signaling to HCMV infection and how these pathways contribute to oncogenesis.

## HCMV and Cancer

HCMV infection commonly causes acute infection in cancer patients undergoing immunosuppressive therapies for the treatment of leukemia, lymphoma, and myeloma (Kuo et al., 2008; El-Cheikh et al., 2013; Tay et al., 2013; Teh et al., 2013). Although controversial, it has also been associated with certain brain cancers including malignant glioma and medulloblastoma (Cobbs et al., 2002, 2007; Baryawno et al., 2011; Soroceanu et al., 2011; Ranganathan et al., 2012; Rahman et al., 2019). Due to the immunosuppressive nature of the treatments used to fight various cancers, these cancer patient populations are highly susceptible to HCMV infection. Not only are they more likely to be affected by acute infection or reactivation of latent infection, but HCMV encodes for gene products with oncogenic potential that could promote cancer formation and further contribute to cancer progression.

HCMV is not considered to be a directly transforming virus, yet many of its gene products are capable of driving specific oncogenic phenotypes. This property, known as oncomodulation, suggests that HCMV infection may play a yet unknown role in oncogenesis by transforming the cellular environment into one more conducive to tumor formation. HCMV infection and its oncogenic potential has been reviewed in detail by many [see Michaelis et al. (2009), Herbein (2018), Nauclér et al. (2019)]. In brief, HCMV infection institutes many host cell changes that mirror the hallmarks of cancer including: manipulation of cellular energy metabolism, promoting the cell cycle, and evading growth suppression, avoiding the immune response while promoting inflammation, cellular immortalization, activating invasion, motility, and angiogenesis, genomic changes, and manipulation of the apoptotic response. HCMV prevalence, indicated by the presence of its nucleic acids or proteins, has been associated with multiple cancer types, including breast, colon, prostate, liver, salivary, brain, and soft tissue cancers (Hanahan and Weinberg, 2011; Nauclér et al., 2019). A recent study has taken a deep look into the transcriptome of 2,658 cancers from 38 tumor types. HCMV was associated at low levels with most of the tumor types, although further validation is needed to better understand if HCMV infection is actually associated with all of the presented tumor types or if it was a contaminate due to its frequency across the samples tested (Zapatka et al., 2020). Additionally, some groups have shown that normal colorectal and breast tissues, adjacent to HCMV-infected tumors, often remain uninfected, further suggesting a specific role for HCMV in the tumor microenvironment (Taher et al., 2013, 2014; Bai et al., 2016).

## Calcium Signaling: CAMKK

**Figure 1:**
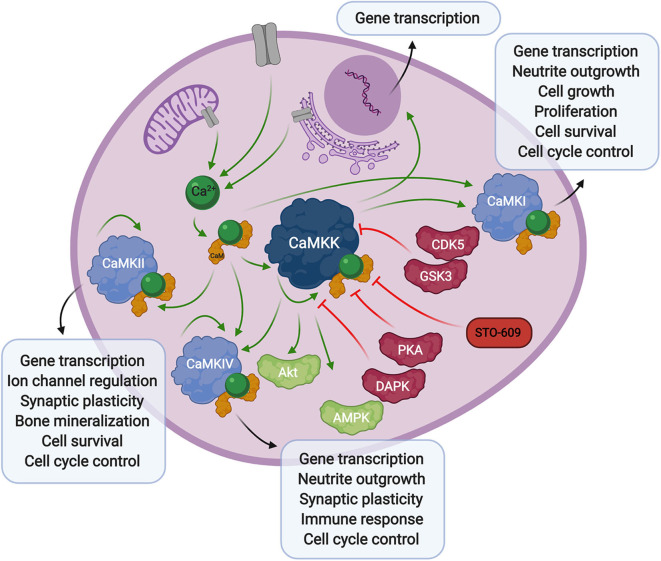
Overview of calcium signaling. Calcium release from the extracellular space or intracellular compartments into the cytosol activates calmodulin, which in turn binds to CaMKK and CaMK proteins. Phosphorylation of CaM-binding proteins via autophosphorylation or upstream kinases further activates, or in some cases inhibits their activity toward downstream signaling proteins and transcription factors. The CaMKK inhibitor, STO-609, can also stall these downstream processes.

Calcium signaling plays a pivotal role during HCMV infection. In this section we will describe calcium signaling orchestrated by a family of serine/threonine kinases and the downstream consequences of calcium signaling (Figure 1). We will also explore the viral proteins and HCMV-induced cellular proteins that contribute to aberrant calcium signaling.

Calcium-calmodulin (Ca^2+^-CaM) dependent protein kinase kinase (CaMKK) is encoded by two genes, CAMKK1 and CAMKK2 resulting in the expression of CaMKKα (or *CaMKK1*) and CaMKKβ (or *CaMKK2*) proteins, originally studied in rat brain (Edelman et al., 1996). CaMKK exists in an autoinhibited state in the presence of basal cellular calcium levels. Calcium flux through the plasma membrane or release of internal stores into the cytoplasm activates the small messenger protein, calmodulin, which in turn releases autoinhibition and activates CaMKK kinase activity toward downstream Ca^2+^-CaM activated proteins, CaMKI (Lee and Edelman, 1994), and CamKIV (Tokumitsu et al., 1994). Regulation of CaMKK via other kinases also plays a role in the Ca^2+^-CaM signaling cascade. The phosphorylation status of CaMKK can determine the fate of Ca^2+^-CaM binding. Specifically, serine-458 phosphorylation facilitated by cAMP-dependent protein kinase (PKA), within the CaM binding domain of CaMKK, will block CaM from binding thus inhibiting CaMKK activity (Matsushita and Nairn, 1999; Davare et al., 2004). More recently, it was also shown that multi-phosphorylation of CaMKKβ by cyclin-dependent kinase 5 (CDK5) and glycogen synthase kinase 3 (GSK3) results in decreased kinase activity and decreased CaMKKβ protein stability (Green et al., 2011). Alternatively, autophosphorylation at threonine-482 in the regulatory domain of CaMKKβ increases its activity by decreasing its autoinhibition, independent of Ca^2+^-CaM binding (Tokumitsu et al., 2001, 2011).

CaMKK activity plays a role in gene transcription through regulation of its downstream CaMK proteins, as well as its activity toward AMPK (Woods et al., 2005) (discussed below). It also interacts with protein kinase B (PKB/Akt) (Yano et al., 1998), via phosphorylation of threonine-308 and serine-473 (Gocher et al., 2017), resulting in an antiapoptotic effect through upregulation of pro-survival pathways. CaMKK activity has also been linked to apoptosis through phosphorylation of CaMKKβ by death associated protein kinase (DAPK), whose activity toward CaMKKβ reduces CaMKK autophosphorylation and elicits a proapoptotic response. The authors suggest this mechanism could occur through reduction of CaMKK phosphorylation of the downstream effector, Akt (Schumacher et al., 2004). These studies show that not only does CaMKK play a critical role in direct Ca^2+^-CaM signaling through CaMKI and CaMKIV, but it is able to influence the apoptotic pathway in a more direct manner.

Expression of CaMKK has been identified in an array of rat tissues, most notably in the brain, and CaMKKβ is also found at lower levels in the thymus, spleen, lung, and testis (Tokumitsu et al., 1995; Anderson et al., 1998). In human tissue, CaMKK is again predominantly found in the brain (Ohmstede et al., 1989), but is widely detected across multiple tissues at the RNA and protein levels, including enrichment of CaMKKα in endocrine, digestive tissue, prostate, bone marrow, lymphoid tissues, and enrichment of CaMKKβ in lung and heart muscle (Uhlén et al., 2015). Additionally, CaMKKβ encodes for distinct isoforms, most notably, CaMKKβ1 and CaMKKβ2, which exhibit differential activities and expression patterns in normal vs. human tumor tissue and cell lines. Most notably, normal human brain tissues express transcripts for CaMKKβ1 and CaMKKβ2, while brain tumors express smaller CaMKKβ1 transcript variants at high levels. Preferential expression of these smaller transcripts was also observed in established brain tumor cell lines (Hsu et al., 2001). It was also shown that exon 14 of CaMKKβ is required for its autophosphorylation, independent of Ca^2+^-CaM binding, but that Ca^2+^-CaM binding is required for its downstream activity toward CaMK proteins (Hsu et al., 2001). Due to the nature of CaMKKβ expression in normal and cancerous brain tissues, its activity could also play a role during HCMV infection. The impact of HCMV infection on the structural development of the brain is discussed in more detail below.

### Calcium Signaling: CaMKI, CaMKII, CaMKKIV

CaMKI exists in four isoforms, CaMKIα, CaMKIβ/Pnck, CaMKIγ/CLICK3, and CaMKIδ/CKLiK, each encoded by a separate gene (*CAMK1, PNCK, CAMK1G*, and *CAMKID*). All contain similar autoinhibitory domains, which require release via phosphorylation by CaMKK and Ca^2+^-CaM binding for full activation (Soderling and Stull, 2001; Senga et al., 2015). It is the most ubiquitously expressed group of CaMK proteins, and the isoforms can be found at various levels in all tissues (Picciotto et al., 1995), but are most highly expressed in brain tissues (Nairn and Greengard, 1987; Kamata et al., 2007). Substrates of CaMKI are generally involved in gene transcription (Swulius and Waxham, 2008). Two widely studied CaMKI targets are extracellular signal-regulated kinase (ERK) and cAMP response element-binding protein (CREB). Predominantly CaMKIα and CaMKIβ activities toward CREB activate neuronal transcription and stimulates neurite outgrowth (Sheng et al., 1991; Yan et al., 2016). CaMKI is responsible for the induction of ERK transcriptional activity, which results in the stimulation of cell growth, proliferation, and cell survival pathways (Schmitt et al., 2004, 2005). There are several other reports of CaMKI activity toward central transcriptional regulators, each having similar outcomes on cell survival and growth with response to calcium signaling.

CaMKII is activated by CaM-binding and autophosphorylation activity. It is responsible for the regulation of many downstream targets involved in ion channel regulation, synaptic plasticity, and gene transcription (Swulius and Waxham, 2008). CaMKII is unique in that it exists as a multimeric dodecamer in the cell, as opposed to the other CaM-binding proteins, which exist in monomeric forms. There are four isoforms, CaMKIIα, CaMKIIβ, CaMKIIγ, and CaMKIIδ (encoded by *CAMK2A, CAMK2B, CAMK2G*, and *CAMK2D*). Each varies slightly in size and contains a variable linker region which can produce splicing isoforms. The isoforms are differentially expressed in multiple tissues throughout the human body (Brzozowski and Skelding, 2019). CaMKIIα and β are primarily expressed in brain tissue, while γ and δ are more ubiquitous across tissue types. The multimeric structure of CaMKII is thought to contribute to regulation of synaptic transmission, as CaMKIIα, β, and γ are commonly associated with synaptic vesicles (Ouimet et al., 1984; Takamori et al., 2006; Wang, 2008). Autophosphorylation of threonine-286 is essential for synaptic plasticity, learning, and memory in the brain (Giese et al., 1998). CaMKII also plays an important role in Ca^2+^ channel activity in heart muscles, where it phosphorylates and tethers itself to channel component, α1c, thereby tightly regulating Ca^2+^ spikes in the heart (Hudmon et al., 2005; Maier and Bers, 2007). During osteoblast differentiation, CaMKII is stimulated by Ca^2+^ influx, and phosphorylates downstream proteins: cAMP-response element (CRE) and serum response element (SRE). CaMKII further facilitates transcriptional changes, often associated with osteoblast differentiation, thus stimulating bone mineralization (Shin et al., 2008). More recently, CaMKII has been shown to be important in the regulation of cell death in both neurons during ischemia via an unknown mechanism (Rostas et al., 2017) and osteoblasts through activation of multiple upstream pathways including ER stress, MAPK activation and mTOR signaling (Liu et al., 2018). CaMKII is a physiologically versatile and extensively studied CaMK protein, which is required for the regulation of multiple cellular functions crucial for normal cellular function and functions important for HCMV infection.

Both CaMKII and CaMKI have also been implicated as important mediators of cell cycle progression. Expression of a kinase-dead CaMKI mutant elicits a stall in the G1 cell cycle phase, while inhibition with CaMKII inhibitors has a negative impact on the G2/M and metaphase-anaphase transitions of the cell cycle (Skelding et al., 2011). The role of CaMKII in microtubule dynamics by regulation of centrosome duplication (Matsumoto and Maller, 2002), may also impact AMPK-mediated modulation of cytoskeletal and microtubule dynamics. Additionally, CaMKII has been linked to mitotic instability (Mones et al., 2014), often associated with cancers.

CaMKIV is activated via CaMKK phosphorylation, but also exhibits autophosphorylation capabilities. These phosphorylation events, in combination with CaM-binding, result in full activation of downstream target proteins. It is encoded by one gene, *CAMK4*, which produces two or more splice variants (Brzozowski and Skelding, 2019). CaMKIV is primarily responsible for an array of gene transcription events and is expressed in the brain at high levels (Ohmstede et al., 1989) but can be found in other cell types and tissues such as immune cells and reproductive organs (Skelding et al., 2011). Transcriptional activity of downstream CaMKIV targets has also been implicated in neurite outgrowth, the immune response and cell cycle control. CaMKIV can activate CREB activity, causing a Ca^2+^ dependent regulation of transcription (Enslen et al., 1994), but to a lesser extent than observed with CaMKI stimulation in most studies (Enslen et al., 1995). CaMIV activation of CREB has been linked to synaptic plasticity (Bleier and Toliver, 2017), has been found to be required for fear memory (Wei et al., 2002), and has been linked to hematopoietic stem cell homeostasis (Kitsos et al., 2005). Cell cycle control through regulation of microtubule dynamics has also been linked to CaMKIV activity (Melander Gradin et al., 1997). Finally, as reviewed here (Racioppi and Means, 2008), CaMIV plays a pivotal role in immune cells and the inflammatory response, leading to the possibility that the status of CaMIV in the cell could modify the permissiveness of a cell to HCMV infection and tumor formation or invasion. In general, CaMKI, CaMKKII, and CaMKIV activities are similar, but their distinct differences could potentially impact the state of a cell during HCMV infection and lead to oncomodulation of the cellular environment.

## Calcium Signaling: Substrate-Specific CaM-Binding Kinases

There are three characterized Ca^2+^-CaM binding proteins that phosphorylate only a single known substrate and are referred to as substrate-specific calcium signaling molecules. CaMKIII, also known as elongation factor 2 kinase (eEF2K), solely requires CaM-binding for its activation (Swulius and Waxham, 2008). It inhibits protein translation by phosphorylating elongation factor 2 (eEF2), thus dissociating it from the ribosome in skin, lung, gastrointestinal, pancreas, reproductive, bone, and lymphoid tissues (Nairn and Palfrey, 1987; Uhlén et al., 2015). Another Ca^2+^-CaM activated kinase, MLCK, acts in muscle contraction (Kamm and Stull, 1985; Word et al., 1994), and intracellular transport in muscle tissue (Mochida et al., 1994), through myosin activation. Finally, Phosphorylase kinase is the only non-monomeric substrate-specific CaM-binding protein, consisting of a complex tetrameric structure, that is ubiquitously expressed across numerous tissue types, but is most commonly found in skeletal muscle and liver tissues (Swulius and Waxham, 2008). It acts toward glycogen metabolism via phosphorylation of glycogen phosphorylase, thereby regulating energy needed for muscle contractions (Kishimoto et al., 1977; Cohen et al., 1980; Picton et al., 1981) and maintenance of blood-glucose levels (Conaglen et al., 1985). A role for the substrate-specific CaM-binding proteins during HCMV infection or in cancer has yet to be elucidated.

## HCMV-Mediated Modulation of Calcium Signaling

Several HCMV genes have been linked to regulation of calcium signaling. Many of these are associated with apoptotic functions, including US21, UL37, and US28. US21 is a viroporin which encodes for a Ca^2+^-permeable channel responsible for decreasing intracellular calcium stores and protects against the intrinsic apoptotic response induced by various drugs (Luganini et al., 2018). UL37 encodes for the viral mitochondria-localized inhibitor of apoptosis (vMIA), which is an inhibitor of the pro-apoptotic protein, Bax, thus promoting cell survival (Goldmacher et al., 1999; Arnoult et al., 2004; Poncet et al., 2004). Additionally, UL37 localizes to both the mitochondrial membrane and the endoplasmic reticulum (ER), where it causes dysregulation in the membrane structure of the mitochondria, and leads to release of calcium stores into the cytosol from the ER (McCormick et al., 2003; Sharon-Friling et al., 2006).

US28, on the other hand, promotes apoptosis through caspase activation, presumably to promote HCMV-associated disease progression in specific cell types (Pleskoff et al., 2005). It is a viral G protein-coupled receptor responsible for internalizing chemokines (Gao and Murphy, 1994). This internalization of chemokines is accompanied by a release of intracellular calcium (Gao and Murphy, 1994; Vieira et al., 1998). Another study examined the effects of US28 expression in a variety of cell types permissive to HCMV infection including smooth muscle, endothelial, and glioblastomas cells. US28 was found to drive phospholipase C-β (PLC-β) which in turn drives intracellular calcium release in all cell types with the exception of glioblastoma cells, in which the authors suggest this could play a role in latent infection. PLC-β signaling was dependent on chemokine response in most cells but could also occur in the absence of chemokines in smooth muscle cells (Miller et al., 2012).

Another viral protein involved in chemokine response, UL146, encodes for a viral chemokine (vCXCL1) which acts as an agonist for human chemokines (CXCR1 and CXCR2) responsible for intracellular calcium release. This chemokine response also has implications in neutrophil recruitment, which then act as passive carriers of the virus. These data suggest that calcium signaling plays a role in viral dissemination through the host (Penfold et al., 1999; Wang et al., 2003). Finally, it is suggested that HCMV binds epidermal growth factor receptor (EGFR) as part of the internalization of the virus, which also causes the mobilization of intracellular calcium, a response that is known to occur with native ligand binding of EGF to EGFR (Wang et al., 2003). Additionally, EGF-mediated calcium release has been linked to cell migration and angiogenesis in glial cells, which again could contribute to HCMV infection in certain brain cancer patients (Bryant et al., 2004). Collectively, these data suggest that multiple HCMV gene products significantly modulate calcium signaling to impact cell growth, apoptosis, and inflammatory responses.

## Contributions of Calcium Signaling to Infection

Activated calcium signaling could contribute to infection in a variety of ways. CaMKK activity plays a crucial role in transcriptional regulation of the cell, largely through downstream activation of CaMKI, CaMKII, and CaMKIV. Activation of these kinases also impacts cellular trafficking, protein translation, apoptosis, metabolism, ion channel regulation, intracellular transport, cell cycle control, and immune cell function, all of which are crucial for HCMV infection. Although this group of proteins is generally enriched in the brain, these proteins are found ubiquitously throughout the human body. Naturally, since the calcium signaling proteins are found at higher levels in the brain, they have been best characterized predominantly in this context. Although lower in other tissues, calcium signaling can still be very important to normal cellular function, and there is evidence to its activity being hijacked by HCMV infection to create a more permissive environment for viral replication. Many proteins are known to be induced by stressful events such as infection, including CaMKKα (McArdle et al., 2011). Induction of CaMKK upon HCMV infection contributes to HCMV-mediated glycolytic activation. Treatment of cells with STO-609, a potent inhibitor of CaMKK, attenuates viral infection and inhibits HCMV-mediated glycolytic activation (McArdle et al., 2011).

As discussed, calcium signaling plays a critical role in the regulation of neuronal cell growth and development. HCMV infection can impact this system in neonates, causing microcephaly and developmental delays at birth. A recent study has shown that HCMV infection impacts the ability of organoid tissues to organize, develop, and differentiate properly. In this study, the authors show that HCMV infected neural progenitor cells lose calcium channel signaling and lose their ability to respond to changes in calcium levels (Sison et al., 2019). The altered state of infected cells overall disrupted structural development of cortical organoids (Sison et al., 2019), which could contribute to the formation of improperly functioning synapses and overall brain mass loss during development leading to HCMV-associated brain impairments in infants.

## HCMV Induction of Calcium Signaling could be Implicated in Tumorigenesis

Many of the same calcium signaling pathways crucial to HCMV infection could also contribute to the oncogenic potential of the cell. Calcium signaling has been the target of many anti-cancer studies due to its importance in cellular processes involved in cancer cell survival and progression. These studies included the use of CaMKK inhibitor STO-609, CaMKII inhibitor KN-62/KN-93, Berbamine, a CaMKIIγ inhibitor, and peptides designed to target CaMKII, all of which are reviewed here (Brzozowski and Skelding, 2019). In general, use of these calcium pathway inhibitors decreased proliferation, migration and invasion, induced apoptosis, or slowed cell cycle progression in multiple cancer cell and tumor types including prostate, medulloblastoma, glioma, lung, breast, and T cell lymphoma, as well as others (Brzozowski and Skelding, 2019). Additionally, multiple studies have suggested that US28 can also promote proliferation and angiogenesis by modulation of inflammatory factors, resulting in enhanced tumorigeneses upon infection of intestinal epithelial cells (Bongers et al., 2010), glioblastoma cells (Slinger et al., 2010), and tumorigenic NIH3T3 cells in mouse models (Maussang et al., 2009; Slinger et al., 2010). Primary glioblastoma tumors from patients were also examined for the presence of US28, which correlated with STAT3 phosphorylation, IL-6 production, higher levels of tumor proliferation, and poorer patient outcomes (Slinger et al., 2010). Finally, CaMKKβ has also been implicated in promoting prostate cancer progression through the upregulation of lipogenesis, a phenotype also associated with HCMV infection (Penfold et al., 2018).

## AMPK Signaling

**Figure 2:**
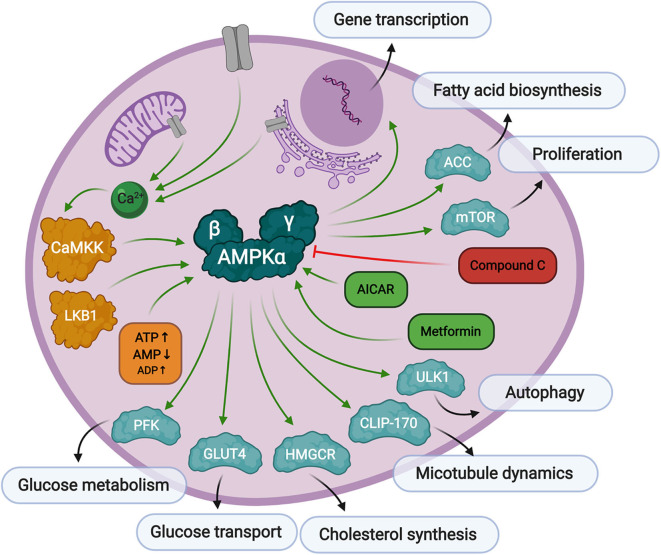
Overview of AMPK signaling. AMPK responds to low levels of ATP in the cell. First, AMP binds to the AMPKγ regulatory subunit causing a conformational change exposing threonine-172 in the AMPKα catalytic subunit. Then, Thr-172 is phosphorylated by upstream kinases, CaMKK, or LKB1, fully activating AMPK kinase activity toward substrates responsible for preserving cellular energy. AMPK can be activated by AICAR or metformin treatment, and inhibited by Compound C.

Similar to CaMKK, AMPK activity has been linked to high HCMV viral titers and HCMV-mediated glycolytic activation. It is also a known tumor suppressor but exhibits some oncogenic phenotypes under specific conditions. In this section we will describe the AMPK structure and function in normal cells (Figure 2), then describe the role it plays in HCMV infection, and how this could contribute to HCMV-associated oncomodulation.

AMPK is a serine/threonine kinase that plays a central role in metabolic stress signaling by responding to low levels of ATP in the cell. The AMPK protein is a heterotrimeric complex consisting of a catalytic subunit, α, and two regulatory subunits, β and γ. There are several protein isoforms of each subunit, α1, α2, β1, β2, γ1, γ2, and γ3, each encoded by their own gene, *PRKAA1, PRKAA2, PRKAB1, PRKAB2, PRKAG1, PRKAG2*, and *PRKAG3*, resulting in 12 possible AMPK complex formations (Ross et al., 2016b). Canonical AMPK activation relies on two signals. The first is the binding of AMP to its regulatory γ domain, which causes a conformational change in the protein heterotrimeric complex to expose threonine-172, thereby promoting its phosphorylation and inhibiting its dephosphorylation by protein phosphatases (Hardie et al., 2016). The secondary phosphorylation step is facilitated by upstream kinases, Liver Kinase B1 (LKB1) (Woods et al., 2003) or calcium signaling protein, CaMKK (Woods et al., 2005), to promote full activation of AMPK. CaMKKβ is known to stimulate AMPK more than CaMKKα (Fujiwara et al., 2016), and can stimulate AMPK activity independently of the cellular AMP/ATP ratios if intracellular calcium levels are elevated in adipocytes (Gormand et al., 2011) and other cell types. It has also been reported that ADP binding to the γ subunit can promote conformational changes making the protein more susceptible to Thr-172 phosphorylation, but to a much lesser extent than AMP binding (Gowans et al., 2013).

The AMPK subunit isoforms exhibit some tissue specificity that can translate into functional differences. In general, the AMPKα1 catalytic subunit is ubiquitously expressed, while the AMPKα2 catalytic subunit is preferentially expressed at higher levels in the liver, skeletal, and cardiac muscles. Additionally, the α2 subunit is not expressed in hematopoietic cells (Stapleton et al., 1996; Foretz et al., 2010a; Wang et al., 2016). This tissue specificity plays a functional role in the skeletal system, where α2 promotes osteogenesis at a higher level than α1 (Wang et al., 2016). Whole mouse knockout of either AMPK catalytic subunit is viable with minor defects at the molecular level (Viollet et al., 2003; Fu et al., 2013a,b). Most notably, α1 knockout mice present with severe anemia due to lack of any AMPK activity in blood cells (Foretz et al., 2010a). Simultaneous knockout of both the AMPKα1 and AMPKα2 subunits, results in embryonic lethality around day 10 in mice (Viollet et al., 2009). Double knockout of the AMPKα subunits, has been successfully preformed in cultured mouse embryonic fibroblast (MEF) cells (Lee et al., 2020). This is not surprising, since AMPK plays an important role in development (Carey et al., 2014; Kaufman and Brown, 2016), but in mature cells it is possible that both catalytic subunits play similar roles, or can at least compensate for one another when they are both present in that cell type. The AMPKβ regulatory subunits are ubiquitously expressed, with a preference for β2 expression in skeletal muscle (Mobbs et al., 2015). Differential roles for the β subunits have not been explored in detail. Finally, it is not well-defined whether the AMPKγ regulatory subunits exhibit tissue specificity, but γ1 is found at higher abundances than γ2 or γ3 in skeletal and cardiac muscles (Pinter et al., 2013). The γ subunit isoforms have also been shown to differentially impact the rate of AMPK activity, where γ2 containing AMPK complexes activate more rapidly than γ1 or γ3 containing AMPK complexes. Additionally, the γ subunits exhibit differential affinities for AMP and ADP which mediate their ability to activate the protein (Ross et al., 2016a; Willows et al., 2017). Again, many of the studies involving AMPK subunit tissue localization have focused on cardiac and skeletal tissues, but this does not preclude AMPK activity from being important for other tissue specific functions in the body, such as the brain where calcium signaling is best characterized.

As mentioned above, AMPK is a stress regulated kinase, which responds to low levels of ATP in the cell, with the overall goal of producing more ATP and rebalancing cellular energy. AMPK inhibits cell growth through inactivation of mTOR signaling (Gwinn et al., 2008; Kalender et al., 2010), and controls autophagy through manipulation of ULK1 (Kim et al., 2011). Its activity is both directly associated with metabolic enzyme modification as well as through more long-term transcriptional regulation of metabolic processes, cell growth, differentiation, immune response, and apoptosis (McGee et al., 2008). Direct glycolytic substrates of AMPK include acetyl-CoA carboxylase (ACC), whose phosphorylation by AMPK inhibits a key step in fatty acid biosynthesis (Park et al., 2002). AMPK also phosphorylates the high capacity glucose transporter, GLUT4, which signals for its translocation to the plasma membrane (Kurth-Kraczek et al., 1999) thus increasing glucose uptake into the cell. Glycolytic flux is also regulated through AMPK phosphorylation of 6-phosphofructo-2-kinase (PFK-2) which controls the levels of 2,6-bisphopsphate, which in turn regulates the activity of the key glycolytic enzyme, 6-phosphofructo-1-kinase (PFK-1) (Marsin et al., 2000). Another role of AMPK is its regulation of cytoskeleton dynamics. As mentioned before, AMPK can be regulated by calcium signaling, which is also a major contributor to microtubule and centrosome structure. AMPK directly phosphorylates CLIP-170, causing dissociation of the growing end of microtubules (Nakano et al., 2010). AMPK has also been implicated in neuronal polarization (Williams et al., 2011). Though not an exhaustive list, it is clear that many of the verified AMPK phospho-targets are generally involved in the preservation and production of cellular energy.

## AMPK Associations With HCMV Infection

Many AMPK targets play a critical role in HCMV infection, including mTOR (Rodríguez-Sánchez et al., 2019), ACC (Spencer et al., 2011), and GLUT4 (Yu et al., 2011). AMPK activity has also been directly linked to HCMV infection. One study preformed an siRNA screen of the cellular kinome, assessing HCMV replication as a readout. In this study, several AMPK subunits, the AMPK activator CaMKK, and several downstream AMPK targets were identified as modulators of HCMV infection. Furthermore, inhibition of AMPK by its inhibitor, Compound C (CC), dramatically changed HCMV-mediated induction of metabolite pools (Terry et al., 2012). Data from *in vitro* AMPK activity assays revealed that HCMV infection activates AMPK. Further, during HCMV infection, AMPKα accumulates and its phosphorylation increases. AMPK activity is necessary for HCMV-mediated activation of glycolysis and production of high viral titers, a process that can be halted by the addition of CC (McArdle et al., 2012). While these studies revealed that AMPK activity is necessary for productive HCMV infection, excessive AMPK activity can also have a detrimental impact on viral production. Use of AMPK activators, AICAR or metformin, also contributes to a loss of viral replication (Kudchodkar et al., 2007; Terry et al., 2012; Li et al., 2017). It has been suggested that timing of AMPK activation plays a critical role in successful HCMV replication. It is possible that AMPK activity is important at a specific time during infection, and less important, or even detrimental at alternate times post infection. It is also possible that the studies utilizing AMPK inhibitors or activators, are observing off-target effects of the drugs themselves. Initially CC was reported as a specific ATP competitive inhibitor through an *in vitro* assay (Hsich et al., 2001). Since then it has been reported that CC can target many more kinases including CaMKK (Bain et al., 2007; Jester et al., 2010). Although metformin is associated with AMPK activation, it does not directly interact with the AMPK protein. Instead it inhibits mitochondrial respiratory chain complex 1, which in turn increases the ratio of AMP to ATP, thus indirectly activating AMPK (Owen et al., 2000). Work has also shown that AMPK does not always play a role in metformin associated phenotypes, as metformin-associated genes remain unchanged between normal, AMPKα knockout, and LKB1 knockout hepatocytes (Foretz et al., 2010b). This suggests a role for metformin that is independent of AMPK activity, which could potentially affect HCMV infection by an unknown mechanism. Excessive AMPK activation could also have a negative impact on its inhibition of certain AMPK targets, such as ACC. AMPK activity toward ACC results in the inhibition of fatty acid biosynthesis, which is detrimental to HCMV infection (Spencer et al., 2011). A similar phenomenon could be occurring when AMPK phosphorylates 3-hydroxy-3-methylglutaryl CoA reductase (HMGCR), which is responsible for cholesterol synthesis, another important cellular process that is key to HCMV growth (Potena et al., 2004; Shenk and Alwine, 2014). It is possible that the virus is able to manipulate ACC and HMGCR activity irrespective of its phosphorylation by AMPK during normal infection but cannot compensate for activated AMPK during AICAR or metformin treatment. Finally, specific combinations of AMPK subunit isoforms may also play a role in AMPK's substrate specificity and ultimately impact HCMV infection but have yet to be fully interrogated.

## AMPK Implications in Cancer

There are reports indicating mutational changes of the AMPK subunits in cancer. First, some evidence suggests that AMPKα1 acts as an oncogene, while α2 may act as a tumor suppressor. The AMPK activator, LKB1, is encoded by the gene *STK11*, which is mutated or deleted in many cancers. Most commonly, in lung carcinomas carrying *STK11* alterations, the AMPKα1 gene (*PRKAA1*) is often amplified, while mutations in the *PRKAA2* gene occur less frequently (Monteverde et al., 2015). In support of this, double knockout of AMPKα1 and α2 in MEF cells transformed with H-RasV12 fail to grow tumors in immunodeficient mice (Laderoute et al., 2006) while transformed MEFS with α2 knockout exhibit a growth advantage in tumors. In contrast, α1 knockouts do not develop tumors and even exhibit compensation with total levels of α2 (Phoenix et al., 2012). These data suggest distinct and separate roles for the AMPK catalytic subunits in cancer, which cannot be rescued with compensation of the alternate isoform. The AMPKβ1 subunit is mutated in <4% of cancers, in contrast to the β2 subunit, which is aberrantly expressed in upwards of 10% of cancers, with its expression commonly amplified (Monteverde et al., 2015). It has also been reported that the α1 subunit requires β2 for its stability during overexpression, which could implicate the correlation between the amplification of these two subunits in cancer (Ross et al., 2016b). There are few reported mutations of the AMPKγ subunit isoforms in cancer. It is possible that their roles change upon mutation or amplification of the α and β subunits.

Many canonical AMPK activities lend themselves to AMPK acting as a tumor suppressor, and therefore loss of expression would promote tumorigenesis (Shackelford and Shaw, 2009). Recent work has shown that treatment of colorectal cancer cells and breast cancer cells and tissues with a novel AMPK activator (FNDs) induces apoptosis and cancer cell death (Kenlan et al., 2017; Johnson et al., 2019). But there is also evidence that AMPK is critical for the maintenance of established tumors and could therefore be targeted for anti-cancer therapies in certain contexts. As mentioned, reports suggest that the AMPKα1 gene acts as an oncogene and the α2 gene acts as a tumor suppressor. AMPKα1 activation via CaMKKβ is reported to promote cancer cell survival and protection against genotoxic stress induced by etoposide treatment, while AMPKα2 did not exhibit this protective effect (Vara-Ciruelos et al., 2018). These data suggest that inhibition of AMPK in AMPKα1 rich tumors may be a more effective treatment. Additionally, tumors requiring a higher metabolic rate may also be disadvantaged by AMPK inhibition and supported by AMPK activation. Studies have shown that tumors require AMPK's metabolic functions to maintain tumor cell viability in the face of energetic stressors (Jeon and Hay, 2012). The same oncogenically-associated characteristics of AMPK activity may also apply to cells infected by HCMV. These data also highlight a prominent role for the AMPK subunits during stressful cellular events requiring functional cell survival and metabolic processes (Jeon and Hay, 2015).

## Intersection of Calcium and AMPK Signaling During HCMV Infection

**Figure 3:**
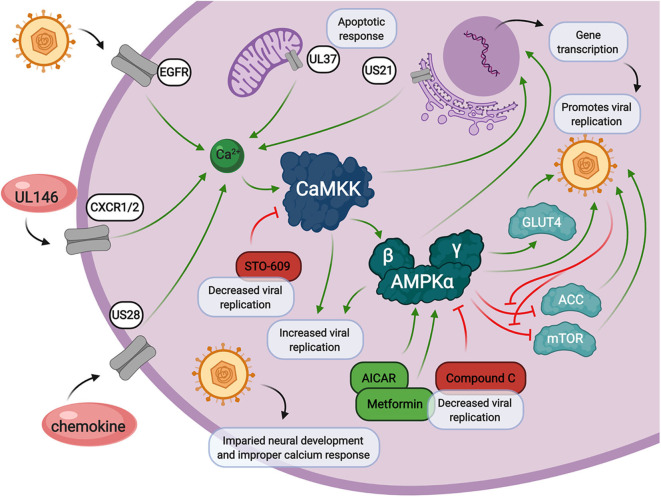
Calcium and AMPK signaling during HCMV infection. Calcium signaling contributes to increased intracellular calcium release through HCMV encoded genes, UL146, US28, UL37, and US21, and through viral entry via epidermal growth factor receptor (EGFR). HCMV infection also induces the expression of CaMKK and AMPK, both of which are crucial for viral replication through multiple cellular signals. HCMV infection also impacts neural cell development which is associated with loss of proper calcium response. Inhibition of CaMKK by STO-609 inhibits HMCV viral replication. AMPK inhibition by Compound C, or activation by metformin or AICAR, also results in a decrease in HCMV viral replication.

As evidenced above, both calcium and AMPK play central roles in the cellular stress response and intersect with each other in the cytosol (Figure 3). Calcium signaling is heavily regulated by viral genes involved in apoptosis and the chemokine response. Upon calcium mobilization from the extracellular space or from within organelles such as the mitochondria or endoplasmic reticulum, CaMKK starts the calcium signaling cascade. This is when AMPK can be activated, in addition to other cellular signals coming from LKB1. Together, CaMKK and AMPK are responsible for immediate phosphorylation signals of target proteins and long-term activation of transcription factors involved in countless cellular processes involved in the promotion of viral replication. Inhibition of either CaMKK by STO-609 or AMPK by CC, as well as activation of AMPK by metformin or AICAR, results in diminished viral replication. These processes are delicately balanced by HCMV infection in order to promote successful release of viral progeny.

## Conclusions and Perspectives

Cellular stress often affects cell proliferation, apoptosis, cell cycle, and the immune response, so it is not unreasonable to think that, in some way, each pathway contributes to modulating host cell homeostasis and HCMV infection. HCMV has adapted to carefully control calcium signaling through many of its gene products, US28, US21, UL37, and UL146, as well as through the manipulation of calcium signaling protein, CaMKK. HCMV also acts downstream of CaMKK, on AMPK, to further manipulate host cell transcription and more specifically, host cell metabolism for the benefit of the virus. There is significant overlap in the downstream consequences of calcium and AMPK signaling, yet the downstream functional targets of these pathways that control HCMV infection are largely unknown. Identifying them and elucidating their contributions to infection will greatly increase our understanding of how these host pathways contribute to infection and could potentially identify targets for therapeutic intervention.

Studies focusing strictly on calcium signaling in the brain and AMPK activity in cardiac or skeletal tissues have opened the door to many unanswered questions about how these signaling pathways contribute to cellular function and various pathologies in other tissues. Many of the proteins discussed above are ubiquitously expressed in all cell types and perform basal functions in normal cells. HCMV infection occurs in a broad range of host cells, and both calcium and AMPK signaling play a significant role during infection. One major question that remains largely unanswered, is how do these signaling pathways contribute to latent HCMV infection? It has been suggested that a lack of calcium signaling, for instance, in the brain may contribute to low lytic infection and inadvertently promote latency in these cells (Miller et al., 2012). Calcium signaling has also been implicated in neutrophil recruitment (Penfold et al., 1999; Wang et al., 2003), which could play a role in the lytic to latent transition. Additionally, AMPKα1 is expressed in hematopoietic cells while AMPKα2 is not (Stapleton et al., 1996; Foretz et al., 2010a; Wang et al., 2016). As major reservoirs for latent virus, studies with hematopoietic and myeloid progenitor cells (Schottstedt et al., 2010; Murray et al., 2018), may highlight an isoform specific role of the AMPK catalytic subunits in HCMV infection or latency.

Lastly, HCMV infection has been associated with numerous types of cancers as discussed above. Many cancers rely on AMPK and calcium signals to promote tumor formation and survival. Although many of these associations are not fully understood, there is evidence that HCMV infection promotes oncomodulation, and this could be mediated by viral modulation of calcium and AMPK signaling. For example, the prevalence of the HCMV gene US28, which is responsible for intracellular calcium mobility (Gao and Murphy, 1994; Vieira et al., 1998), is associated with patient glioblastomas and poor cancer prognosis (Slinger et al., 2010). It remains unknown whether calcium or AMPK signaling are viable targets for anti-HCMV or anti-cancer therapeutics. Based on our current knowledge, and the known overlap between the pathway signaling molecules, there is a possibility that specific CaM-binding proteins, or specific AMPK subunits could be successfully targeted. Future research will further our understanding of the functional interactions between HCMV and these important cellular pathways and will shed light on the potential for targeting these pathways to limit HCMV-associated pathogenesis.

## Author Contributions

DD wrote the manuscript and created the figures. DD and JM contributed to the content and editing of this manuscript. All authors contributed to the article and approved the submitted version.

## Conflict of Interest

The authors declare that the research was conducted in the absence of any commercial or financial relationships that could be construed as a potential conflict of interest.
